# Biomechanical evaluation of carpal joint ligaments in the cat: An experimental and radiographic cadaveric study

**DOI:** 10.1111/vsu.70114

**Published:** 2026-04-28

**Authors:** Mario Candela Andrade, Phillip Meinck, Pavel Slunsky, Leo Brunnberg

**Affiliations:** ^1^ Department of Medicine Health and Medical University Potsdam Brandenburg Germany; ^2^ Small Animal Practice Herzlake Herzlake Lower Saxony Germany; ^3^ Department of Veterinary Clinical Sciences Jockey Club College of Veterinary Medicine and Life Sciences, City University of Hong Kong Hong Kong Special Administrative Region China; ^4^ Small Animal Clinic, Department of Veterinary Medicine Freie Universität Berlin Berlin Germany

## Abstract

**Objective:**

To define the stabilizing role of feline carpal ligaments through selective transection and radiographic assessment of joint angulation under standardized loading.

**Study design:**

Experimental ex vivo cadaveric study.

**Sample population:**

Sixty carpal joints from 30 adult cats without orthopedic disease.

**Methods:**

Carpi were mounted in a custom radiolucent clamping device and subjected to standardized 20 N varus, valgus, and extension loads. Measurement tolerances were 3.5° for varus, 6.4° for valgus, and 6° for extension. Individual ligaments or ligament complexes were sequentially transected, and stability changes were quantified radiographically using dorsopalmar and mediolateral projections.

**Results:**

Transection of the medial collateral ligament produced only minor valgus increase below tolerance thresholds. Palmar radiocarpal ligament desmotomy caused valgus and hyperextension instability with consistent radiocarpal subluxation. Additional flexor retinaculum transection increased extension by up to 27.9° and resulted in antebrachiocarpal luxation. Lateral collateral ligament transection produced marked varus instability (+16.8°) with ulnocarpal subluxation, and the ulnopalmar and accessory ulnar ligaments acted as secondary stabilizers. Distal palmar carpometacarpal ligament transection caused hyperextension (+12.3°) with mediocarpal and carpometacarpal subluxation. Isolated flexor myotomy or tenotomy did not produce measurable instability unless fascial structures were disrupted.

**Conclusion:**

Feline carpal stability depends on coordinated ligamentous and fascial interactions rather than a single dominant stabilizer. This reproducible multiplanar testing model provides a biomechanical basis to improve diagnosis and treatment of feline carpal injuries, which are often extrapolated from canine data.

**Clinical relevance:**

These data highlight that isolated ligament repair may not adequately restore carpal stability in cats. Clinicians should consider the integrated role of ligamentous and fascial structures when diagnosing and managing carpal injuries.

## 1 INTRODUCTION

The carpal joint is essential for forelimb function in carnivores, with mobility reflecting species‐specific locomotor demands.^1^ Canine carpal anatomy, ligament injuries, and treatment have been well described,^2^ but information on cats is limited and restricted largely to isolated clinical reports and experimental studies.^3^, ^4^, ^5^, ^6^ As a result, clinicians often extrapolate from dogs to cats despite known anatomical and biomechanical differences.^7^, ^8^

In dogs, experimental desmotomy has clarified the stabilizing roles of individual carpal ligaments, with Wernitz^9^ providing a systematic biomechanical framework for diagnosis and treatment. Comparable data in cats remain scarce. Voss et al.^5^ showed that even complete transection of the medial collateral ligament may not produce radiographic instability, highlighting diagnostic challenges. Although advanced techniques such as computed tomography arthrography (CTA) and magnetic resonance arthrography (MRA)^6^ and finite element analysis^10^ have been described, the biomechanical effects of selective ligament transection in the feline carpus have not been evaluated experimentally. Similar models exist for the feline tarsus,^11^ but equivalent studies of the carpus—particularly of palmar stabilizers—are lacking.

Carpal injuries in cats are uncommon.^4^, ^12^ Over a 10 year period, Candela Andrade et al.^16^ reported 44 cases among 1125 cats with high‐rise syndrome, most affecting the carpometacarpal and antebrachiocarpal joints. However, clinical reports rarely specify the ligamentous, capsular, or muscular structures involved. This gap in species‐specific biomechanical data highlights the need for controlled analysis of feline carpal stability. This study radiographically quantified angular changes following targeted desmotomy and fasciotomy to address this gap.

The aim of this study was to assess feline carpal stability radiographically and quantify angular changes following targeted desmotomy and fasciotomy using a standardized clamping device. It was hypothesized that ligamentous structures contribute unequally to carpal stability, with palmar structures playing a dominant role.

## 2 MATERIALS AND METHODS

Investigations were performed on carpal joints of cats euthanized or deceased for reasons unrelated to this study between October 2013 and March 2015 at the Small Animal Teaching Hospital of the Freie Universität Berlin (Berlin, Germany), with written owner consent for research use. Angular deviations under a constant 20 N load in varus/valgus and extension were measured radiographically in intact and iatrogenically ligament‐injured carpal joints using a before–after design (intact vs. desmotomized). Only skeletally mature cats (≥3 years) were included. Due to limited specimen availability, age and weight were not standardized.

Medical records were reviewed to determine cause of death, and cases were excluded if underlying diseases could affect bone quality or the musculoskeletal system. Exclusion criteria included systemic metabolic disease, inflammatory or infectious conditions, neoplasia, or localized musculoskeletal, vascular, or neurological disorders affecting the forelimb. Anatomical data, clinical records, and blood test results were reviewed, and limbs were radiographically examined in standard views by a certified radiologist (LB). Specimens with radiographically detectable soft tissue or skeletal abnormalities were excluded. Forelimbs without abnormalities were amputated at the scapulohumeral joint following Fossum^13^ guidelines; the remaining bodies were transferred to the Institute of Veterinary Pathology of the Freie Universität Berlin (Berlin, Germany) for further use or disposal.

### 2.1 Amputation and preparation of the forelimbs

Forelimbs were dissected free of skin and subcutaneous tissue, wrapped in gauze soaked in Ringer's solution, and frozen at −20°C.^14^ Before biomechanical testing, limbs were thawed at room temperature for 24 h, joints were gently mobilized, and tissues were examined by palpation and radiography for storage damage.

To ensure reproducible positioning, the antebrachium was secured in a clamping device and the metacarpal bones stabilized with three cerclage wires: one encircling metacarpals I–III, one around IV–V, and one encompassing I–V with twisted ends (Figure 1). A polypropylene string looped laterally and medially through the distal cerclage enabled alignment of the joint within the clamp. Radiographs were obtained at 45°, 90°, and 135° to visualize all but the palmar aspect (Supporting Information, Figure S1).

**FIGURE 1:**
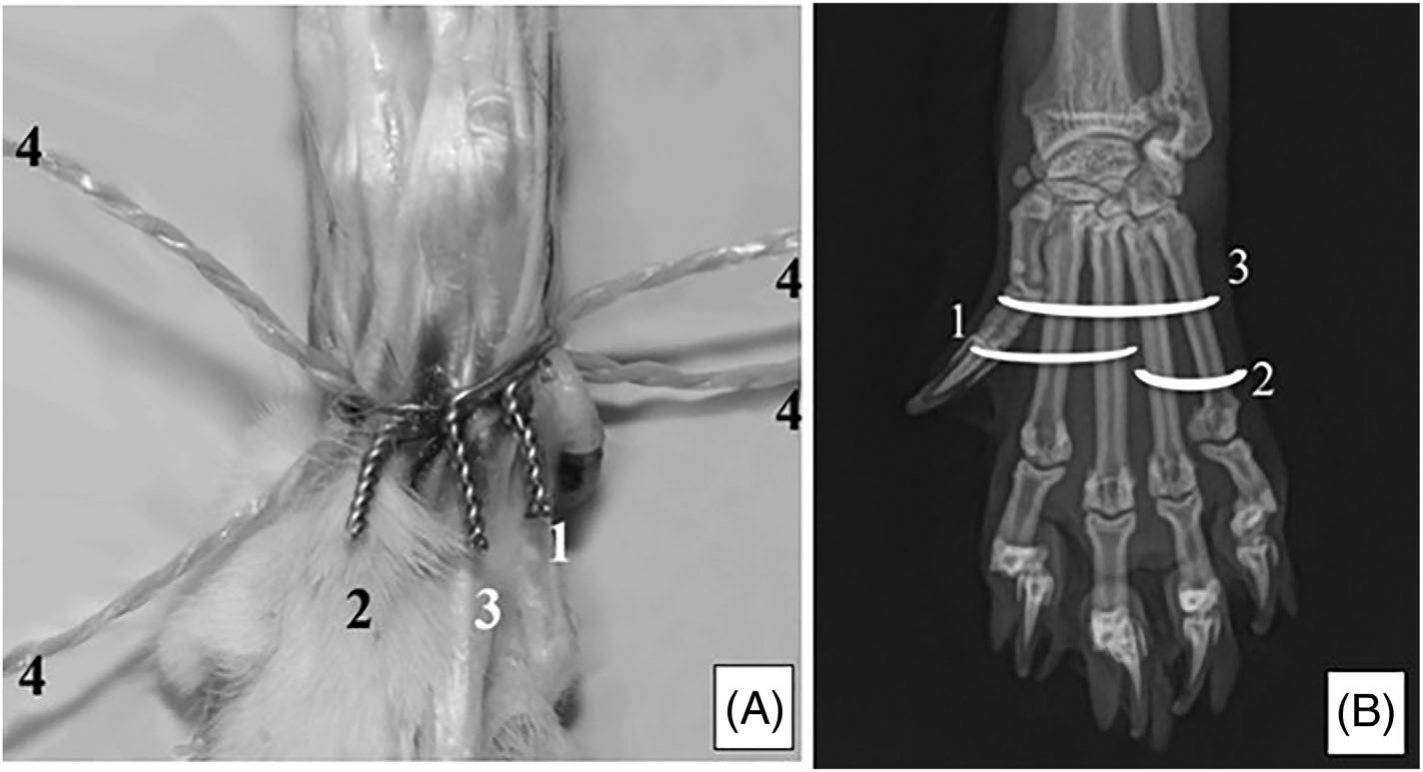
(A) Configuration of the wire cerclages that hold the metacarpal bones in position. (B) Radiograph of a left carpus. The positions of the three wire cerclages on the metacarpus are shown schematically. 1—Cerclage around metacarpal bones I–III. 2—Cerclage around IV–V. 3—Cerclage around I–V. 4—Polypropylene packing string.

### 2.2 Device construction and setup

Carpal stability testing required a custom‐made positioning device ensuring reproducible loading and imaging. To avoid radiographic artifacts, all components within the imaging field were constructed from nonmetallic materials. Based on a preliminary pilot trial performed on 10 forearms, 20 N was identified as the maximal load producing reproducible joint displacement while maintaining mechanical stability of the specimens and testing apparatus.

The device consisted of two parallel Plexiglas plates (34.5 × 28 cm; 4 mm thick each; total thickness 8 mm) joined by eight wing screws, with a 13 cm central groove for the antebrachium. Adjustable clamping units (parts 4/4′, 5/5′) fixed the scapula, humerus, radius, and ulna. Unit 4/4′ held the proximal limb at a 10° angle for scapular alignment, and unit 5/5′ stabilized the forearm using trapezoidal recesses (3.5 × 1.5 × 0.5 cm or 2.5 × 1 × 0.5 cm) cast from Candulor Aesthetic Prosthetic (opaque dark pink, I‐027023; CANDULOR AG, Glattpark (Opfikon), Switzerland) (Figure 2).

**FIGURE 2:**
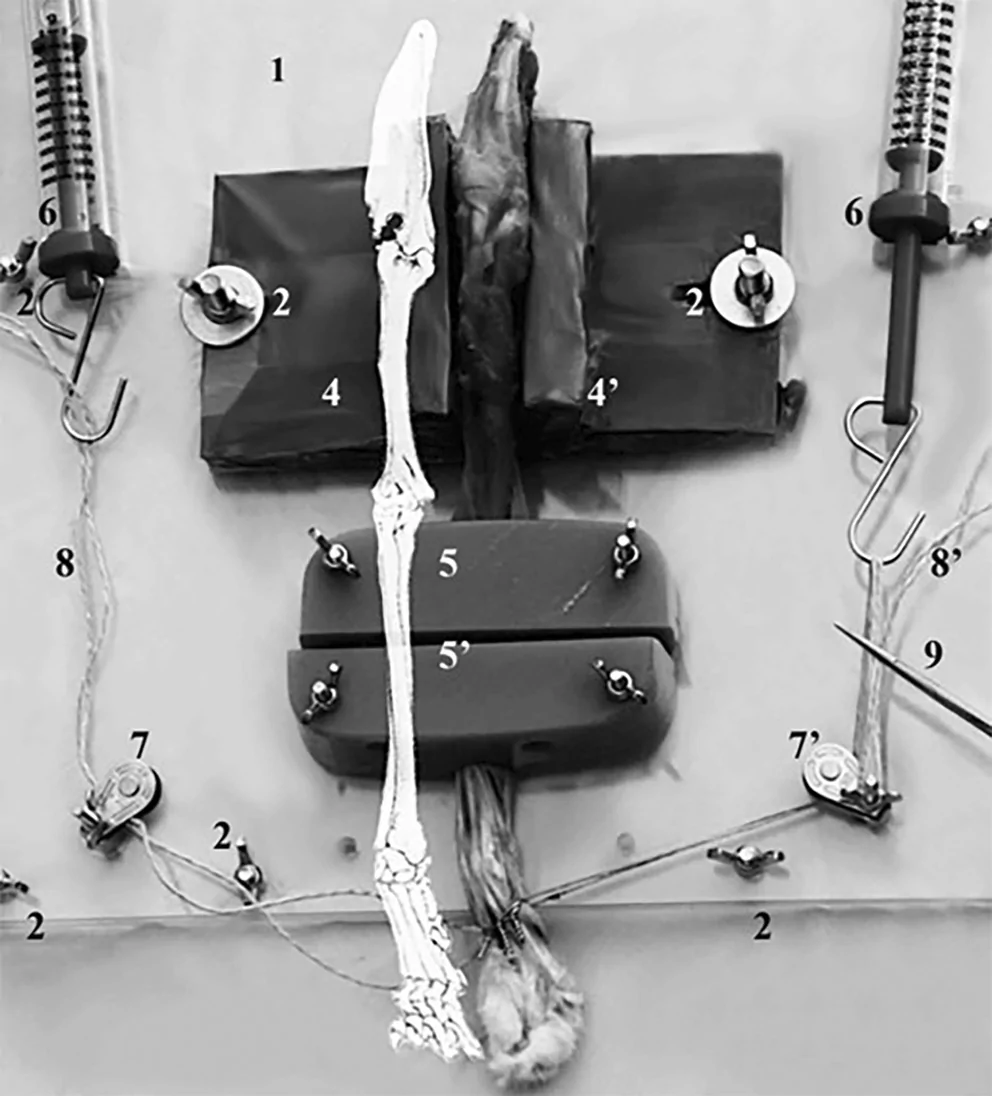
Clamping device in dorsopalmar configuration with the limb secured and the carpal joint in varus. The drawn forelimb illustrates the positioning of the joints on the device. 1—Plexiglas plate. 2—Wing screws. 4/4′—Clamping unit for scapula and humerus. 5/5′—Clamping unit for radius and ulna. 6/6′—Force gauges. 7/7′—Pulley. 8/8′—Package string. 9—Artery clamp.

Force gauges (20 N; parts 6/6′) mounted on the plate edges operated via pulleys (7/7′) and polypropylene strings (8/8′) attached to metacarpal cerclages, transmitting defined loads for varus, valgus, or hyperextension (Figure 3). Tension was maintained with an artery clamp.^9^ For mediolateral stress testing, the orientation of components 5/5′ was modified so that tightening the screws drew the elements together, stretching the limb at the elbow and securing it with the joint positioned between the tension screws. Slight tightening flexed the Plexiglas plates, improving contact without damaging the specimen, following the double‐prism principle^15^ (Supporting Information, Figure S2).

**FIGURE 3:**
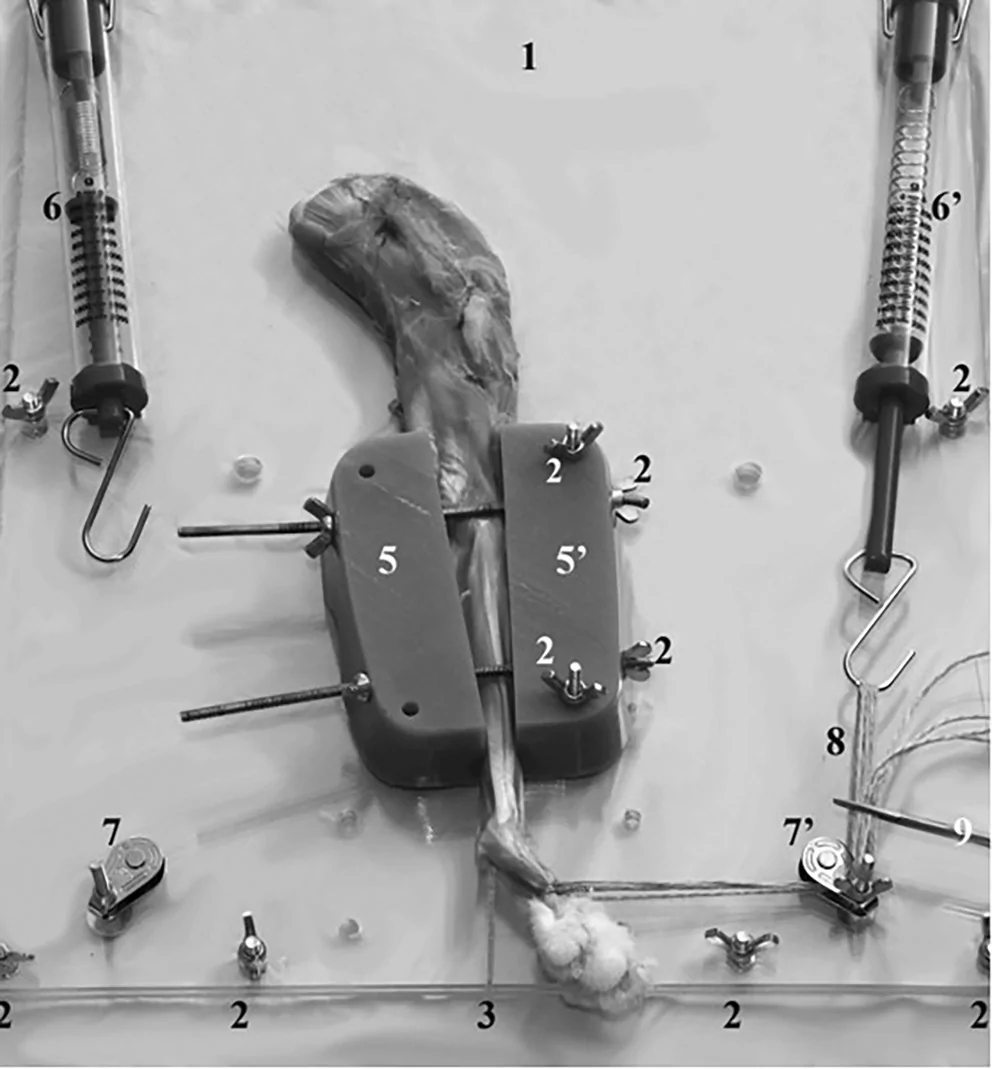
Clamping device in mediolateral configuration, with the limb secured and the carpal joint in hyperextension. 1—Plexiglas plate. 2—Wing screws. 3—Groove. 4/4′—Clamping unit for scapula and humerus. 5/5′—Clamping unit for radius and ulna. 6/6′—Force gauges. 7/7′—Pulley. 8/8′—Package string. 9—Artery clamp.

### 2.3 Testing the clamping device and developing the measurement methodology

Intact amputated forelimbs were radiographed in standard dorsopalmar and mediolateral projections. Additional stress views were obtained in varus, valgus, extension, and hyperextension before and after targeted desmotomy of the respective ligaments. Joint stability was evaluated by comparing predesmotomy and postdesmotomy images.

Slight widening of the joint space with preserved articular congruency was defined as subluxation, whereas marked joint space opening with loss of articular congruency and angular deviation was classified as luxation as described by Allan and Nicoll.^16^

Limbs were radiographed digitally and grid free at 55 kV and 5.0 mAs. Images were saved in DICOM format and analyzed using a RadiAnt DICOM viewer, version 1.9.16.7446 (Poznań, Poland).

The extension angle was measured using a standard goniometer (Figure 4). Reference points for the radial axis were the dorsal edge of the distal articular surface of the radius and the dorsal border of the caput radii. The second arm of the goniometer was aligned tangentially with the dorsal border of the metacarpal bones.

**FIGURE 4:**
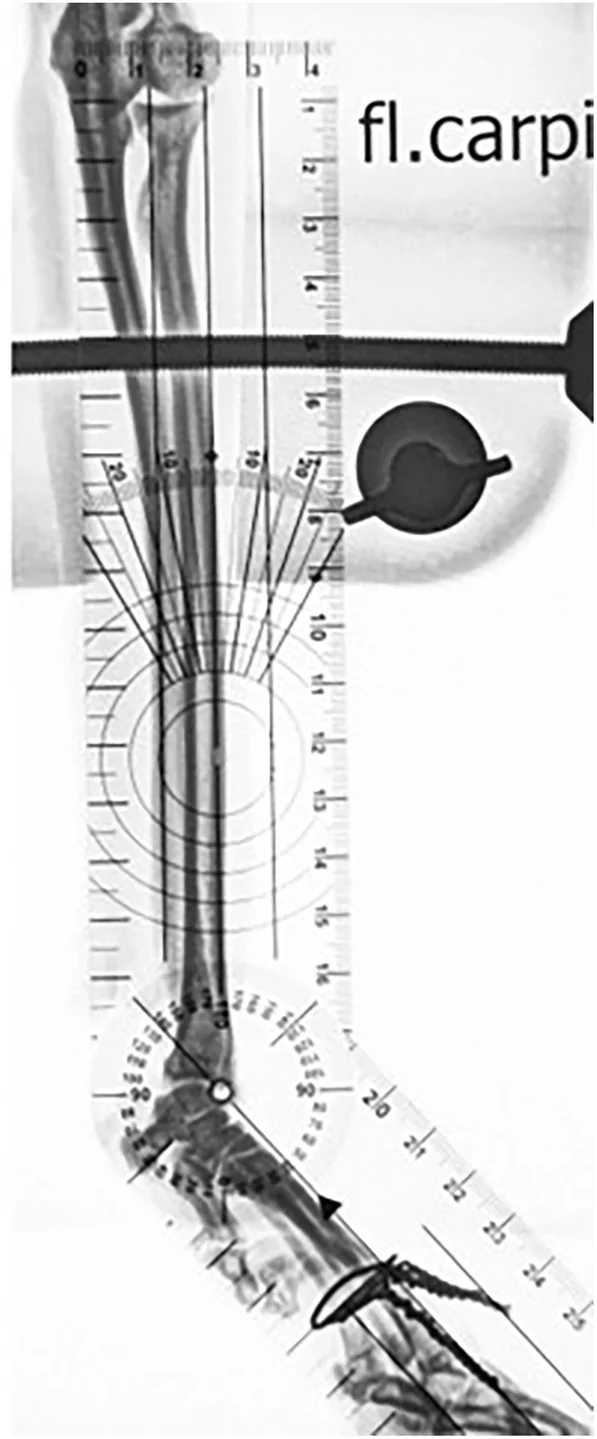
Limb in mediolateral beam path with goniometer used to measure the extension angle. The pivot is placed on distal radius; one arm aligns with the proximal radius, and the other is tangent to the metacarpals.

Joint stability was assessed on dorsopalmar and mediolateral radiographs using reproducible bony landmarks as described by Boone et al.^17^ and Kapatkin et al.^18^ Measurements were performed in standard and stress positions before and after desmotomy. Each measurement was repeated three times per limb by four independent investigators to assess reproducibility.

### 2.4 Preparation of the carpal joint ligaments

For the experimental investigations in the main study, the carpal ligaments were prepared and transected as described by Roos et al.^1^

Palmar carpal ligaments were accessed by dissecting the overlying flexor musculature. The medial and lateral collateral ligaments, palmar ulnocarpal ligament, accessory ulnar ligament, and dorsal radiocarpal ligament were accessible directly after incision of the subcutaneous and fascial tissues.

The following ligaments and ligament combinations were sectioned (Figure 5A,B): flexor carpi radialis and flexor carpi ulnaris; flexor digitorum communis with its tendon sheath and the palmar retinaculum (Figure 5C); and combined transection of the flexor digitorum communis, its tendon sheath, the palmar retinaculum, and the medial collateral ligament.

**FIGURE 5:**
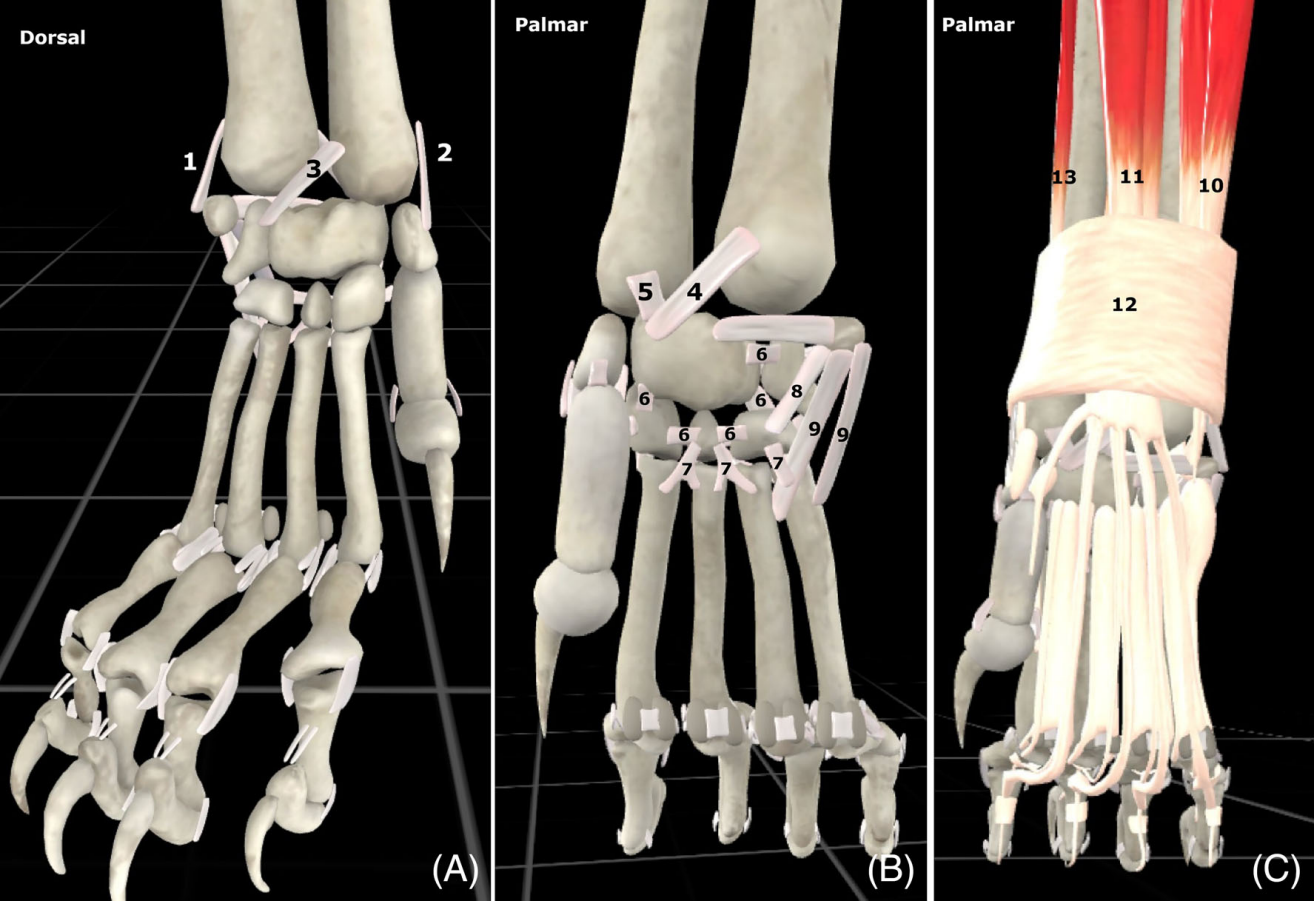
Three dimensional (3D) representation of the transected ligaments and muscles. Image adapted from the Animal Anatomy XR app. Virtual Medicine is the exclusive developer of this product. (A) Dorsal view: 1. Lateral collateral ligament. 2. Medial collateral ligament. 3. Dorsal radiocarpal ligament. (B) Palmar view: 4. Palmar ulnocarpal ligament. 5. Palmar radiocarpal ligament. 6. Palmar intercarpal ligaments. 7. Palmar carpometacarpal ligaments. 8. Accessorioquartile ligament. 9. Accessoriometacarpal ligament. (C) Palmar view: 10. Flexor carpi ulnaris. 11. Flexor digitorum superficialis. 12. Flexor retinaculum. 13. Flexor carpi radialis.

### 2.5 Statistics

Statistical evaluation included data from the preliminary and main experiments. The clamping device and measurement protocol were validated using the coefficient of variation, which was required to remain below 6%. Measurement reliability was assessed by intraobserver repeatability and interobserver reproducibility through repeated measurements and independent examiners. Mean values and maximum deviations were analyzed to confirm compliance with the predefined error margin.

Carpal stability between intact and desmotomized conditions was analyzed descriptively and compared using the Wilcoxon signed rank test for paired samples. Groups with insufficient sample size (*n* < 4) were excluded. Analyses were performed using SPSS Statistics, (v. 21 (Armonk, New York, USA), with significance set at *p* ≤ .05.

## 3 RESULTS

Sixty carpal joints from 30 cats were included in the study. Of the 30 cats, 14 were European shorthairs, one was a European shorthair mix, three were British shorthairs, one a British shorthair mix, and two each were sacred Birman, Persian, and Siamese. The remaining cats represented other breeds. The cats had an average body weight of 4.04 kg (ranging from 2.5 to 5.9 kg) and an average age of 10.8 years (ranging from 3 to 18 years). All cats were neutered—14 female and 16 male (Table 1).

**TABLE 1:** Cat sample characteristics and epidemiological information. All cats were neutered or spayed and varus–valgus range of motion and maximum extension of intact feline carpal joints. Values in parentheses indicate the limb number.

Cat No.	Breed	Age (years)	Sex	Body weight (kg)	Varus–valgus (right) (°)	Varus–valgus (left) (°)	Extension (right) (°)	Extension (left) (°)
1	European shorthair	18	f	3.0	41.6 (1)	43.5 (2)	36 (1)	40 (2)
2	European shorthair	13	f	3.2	50.9 (3)	48.0 (4)	20 (3)	32 (4)
3	European shorthair	6	f	4.6	35.8 (5)	43.4 (6)	No image (5)	No image (6)
4	European shorthair	15	m	2.9	28.7 (7)	31.0 (8)	20 (7)	26 (8)
5	European shorthair	10	m	3.5	28.5 (9)	27.7 (10)	22 (9)	22 (10)
6	European shorthair	12	m	4.7	21.6 (11)	22.7 (12)	20 (11)	20 (12)
7	European shorthair	10	m	5.2	30.8 (13)	29.4 (14)	26 (13)	22 (14)
8	European shorthair	7	f	3.3	34.0 (15)	34.3 (16)	26 (15)	30 (16)
9	European shorthair	14	f	4.3	16.2 (17)	14.7 (18)	20 (17)	24 (18)
10	European shorthair	12	f	3.0	37.5 (19)	41.6 (20)	30 (19)	36 (20)
11	European shorthair	6	m	3.7	27.3 (21)	32.8 (22)	20 (21)	22 (22)
12	European shorthair	15	m	5.0	31.7 (23)	35.7 (24)	30 (23)	30 (24)
13	European shorthair	14	m	5.6	40.4 (25)	34.3 (26)	16 (25)	22 (26)
14	European shorthair	15	m	5.0	37.9 (27)	34.0 (28)	24 (27)	26 (28)
15	European shorthair mix	11	f	5.9	45.2 (29)	37.9 (30)	34 (29)	34 (30)
16	British shorthair	12	m	5.5	32.2 (31)	32.6 (32)	No image (31)	No image (32)
17	British shorthair	7	m	5.4	40.3 (33)	27.0 (34)	28 (33)	30 (34)
18	British shorthair	10	m	5.3	29.0 (35)	26.8 (36)	22 (35)	26 (36)
19	British shorthair mix	3	f	4.1	38.3 (37)	32.0 (38)	26 (37)	22 (38)
20	Sacred Birman	9	m	3.1	50.1 (39)	45.4 (40)	34 (39)	40 (40)
21	Sacred Birman	9	f	3.6	27.3 (41)	33.4 (42)	24 (41)	26 (42)
22	Persian	5	m	4.6	43.0 (43)	44.0 (44)	26 (43)	24 (44)
23	Persian	16	f	3.0	37.8 (45)	33.3 (46)	32 (45)	34 (46)
24	Siamese	12	m	3.4	26.1 (47)	40.4 (48)	22 (47)	24 (48)
25	Siamese	14	f	4.3	25.3 (49)	28.5 (50)	22 (49)	20 (50)
26	Turkish Angora	7	f	3.2	40.4 (51)	37.7 (52)	26 (51)	32 (52)
27	Angora mix	6	m	3.9	29.8 (53)	34.5 (54)	24 (53)	26 (54)
28	Maine coon mix	18	f	2.5	32.8 (55)	34.9 (56)	38 (55)	42 (56)
29	Chartreux	9	f	3.5	34.3 (57)	34.4 (58)	22 (57)	24 (58)
30	Russian blue	10	m	2.9	31.8 (59)	26.9 (60)	16 (59)	20 (60)

Abbreviations: f, female; m, male.

### 3.1 Intact carpal joints

As Table 1 shows, the varus–valgus range of motion of intact carpal joints was 14.7°–50.9°, averaging 34.12° (34.22° right, 34.03° left).

Extension angles (Table 1), measured from a neutral 180° position, ranged from 16°–42°. Mean values were 25.21° (right) and 27.71° (left), yielding an overall mean of 26.46°.

Interobserver and intraobserver variability remained below 6% (mean coefficient of variation 0.43%), confirming high reproducibility. Joint instability was assessed by comparing displacements between standard and stress radiographs before and after desmotomy. Thresholds distinguishing true instability from measurement variation were 3.5° (varus), 6.4° (valgus), 6.0° (extension), and 9.9° (combined range). Displacements exceeding these values indicated radiologically detectable instability.

European Shorthair cats showed a mean varus–valgus range of 33.97°, compared with 34.34° in other breeds. Mean extension was 26.07° in European shorthairs and 26.85° in others. The varus–valgus range was 30.15° in cats under 5 years, 36.46° at 6–10 years, 31.81° at 11–15 years, and 35.24° in older cats, with *p* > .05.

### 3.2 Biomechanical effects of carpal desmotomies

A summary of the biomechanical results for all desmotomies is provided in Figure 6, Supporting Information, Tables S1 and S2, and Table 2. The data show mean changes in varus, valgus, and extension angles before and after transection of individual stabilizing structures of the feline carpus. Each line represents one structure, allowing comparison of their contributions to joint stability. Significant differences are indicated at *p* < .05.

**FIGURE 6:**
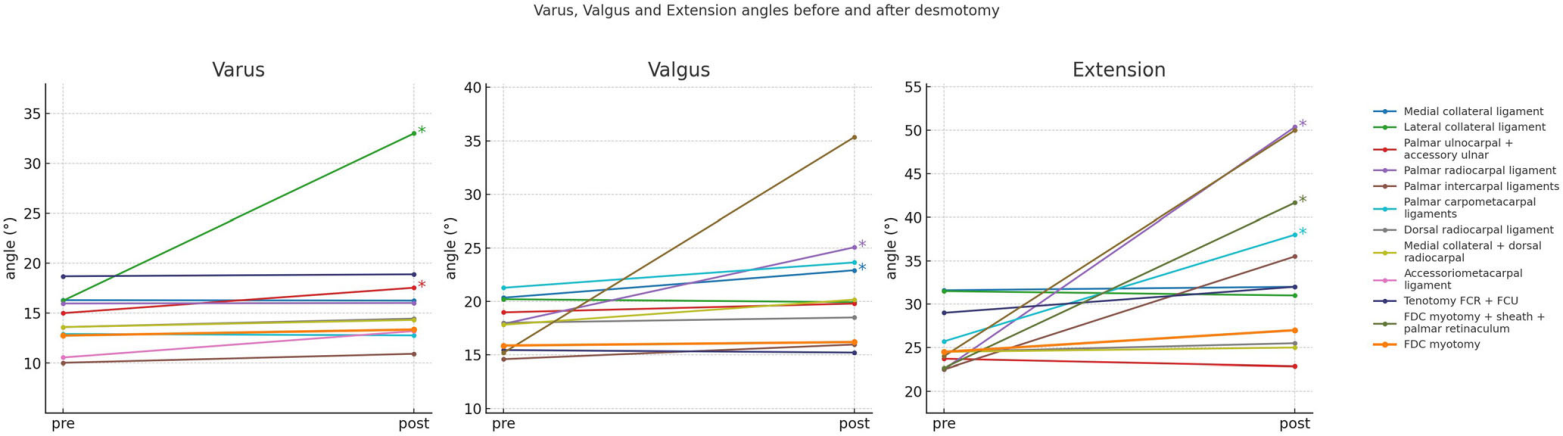
Mean varus, valgus, and extension angles before and after transection of each structure. Asterisks indicate significant differences (*p* < .05).

**TABLE 2:** Mean angular changes after selective desmotomy of feline carpal stabilizing structures.

Ligament/procedure	*n*	Δ Varus (°)	Δ Valgus (°)	Δ Extension (°)
Medial collateral ligament	12	0	+3	0
Lateral collateral ligament	6	**+17**	0	0
Palmar ulnocarpal and accessory ulnar ligaments	7	+3	+1	0
Palmar radiocarpal ligament	8	0	**+7**	**+28**
Palmar intercarpal ligaments	4	+1	+1	**+13**
Palmar carpometacarpal ligaments	7	0	+2	**+12**
Dorsal radiocarpal ligament	4	+1	+1	+1
Medial collateral + dorsal radiocarpal ligaments	4	+1	+2	+1
Accessoriometacarpal ligament	2	+3	+2	+5
Flexor digitorum communis myotomy	4	+1	0	+3
FCR + FCU tenotomy	4	0	0	+3
FDC myotomy + sheath + palmar retinaculum	6	+1	0	**+19**
Medial collateral + retinaculum complex	2	—	**+20**	**+26**

*Note*: Bold denotes clinically relevant instability; *p* values in bold (or <.05) indicate statistically significant differences.

Abbreviations: FCR, flexor carpi radialis; FCU, flexor carpi ulnaris; FDC, flexor digitorum communis.

To access the palmar radiocarpal ligament, the flexor digitorum communis tendon and flexor retinaculum were sequentially transected, and their effects on stability assessed. Transection of the common digital flexor tendons alone did not produce measurable instability. In contrast, sectioning the flexor retinaculum increased instability (mean extension +19°, *p* = .005). Additional transection of the palmar radiocarpal ligament further increased instability, which became most pronounced after combined transection with the medial collateral ligament, resulting in a mean extension increase of 27.88° and antebrachiocarpal luxation (Table 3).

**TABLE 3:** Radiological findings after desmotomy of ligaments and retinacula, tenotomy of selected tendons, and myotomy of palmar forearm muscles stabilizing the carpal joint.

Radiological finding	Structure responsible (after desmotomy)	Varus	Neutral (0°)	Valgus	Extension
Ulnocarpal subluxation	Lateral collateral ligament; palmar ulnocarpal and accessory ulnar ligaments	+	(+)	+	−
Radiocarpal subluxation	Palmar radiocarpal ligament; medial collateral ligament; palmar retinaculum	+	+	+	+
Antebrachiocarpal subluxation	Palmar radiocarpal ligament; medial collateral ligament; palmar retinaculum	−	−	−	+
Antebrachiocarpal luxation	Medial collateral ligament and palmar radiocarpal ligament (combined)	−	−	−	+
Mediocarpal subluxation	Palmar intercarpal ligaments; palmar carpometacarpal ligaments; palmar retinaculum	−	+	+	−
Accessorioulnar luxation	Palmar intercarpal ligaments	+	+	+	−
Carpometacarpal subluxation	Palmar carpometacarpal ligaments	+	+	+	−
Carpometacarpal luxation	Palmar carpometacarpal ligaments	−	−	−	+
Carpometacarpal hyperextension	Palmar carpometacarpal ligaments	−	−	−	+
General carpal hyperextension	Palmar retinaculum; palmar radiocarpal ligament	−	−	−	+

*Note*: +, present; –, absent; (+), occasional/inconsistent finding.

Due to dense fiber integration, individual palmar intercarpal and carpometacarpal ligaments could not be isolated. Instead, the flexor retinaculum, associated palmar ligaments, and joint capsule were transected together; at the middle carpal level, this included the palmar intercarpal ligament complex (including fibers described as the accessorioquartile ligament). Isolated transection at the accessory carpal level did not cause gross instability, which occurred only after combined transection at the middle carpal level.

Desmotomy of the ulnopalmar and accessorioulnar ligaments (transected together due to close association) did not produce measurable varus instability, although radiographic subluxation of the ulnocarpal joint was observed. Extension instability occurred only after additional transection of the lateral collateral ligament and could not be quantified angularly.

Transection of the medial collateral ligament alone did not result in radiographic or goniometric instability. In contrast, lateral collateral ligament transection caused marked dynamic instability, with ulnocarpal subluxation and a mean varus increase of 16.83°.

Palmar intercarpal transection caused middle carpal subluxation, accessory carpal bone luxation, and hyperextension under load. Distal palmar ligament transection resulted in marked hyperextension in 5/7 limbs, while two limbs from the same specimen remained stable, likely due to incomplete transection. Instability extended proximally, with extension increases of 13° at the middle carpal level and 12.29° distally. Isolated transection of the accessory metacarpal ligament, accessory ulnar ligament, or adjacent muscles did not destabilize the accessory carpal joint, whereas common digital flexor muscle and flexor retinaculum transection alone increased extension by 19°.

3.3

Table 3 summarizes the radiologically confirmed results of all experimental procedures for clarity.

## 4 DISCUSSION

This study evaluated feline carpal stability experimentally and radiographically under defined biomechanical conditions in intact and selectively injured forelimbs. Sequential transection of ligamentous and fascial structures identified their stabilizing roles. The results support the hypothesis that these structures contribute unequally to stability, with palmar elements playing a dominant role. The palmar radiocarpal ligament and flexor retinaculum were key proximal stabilizers, as their loss caused marked hyperextension and subluxation. Lateral stability depended primarily on the lateral collateral ligament. The accessorioquartal and palmar carpometacarpal ligaments maintained accessory carpal bone and distal joint integrity.

Carpal injuries in cats are uncommon^4^, ^12^ and are most often reported as carpometacarpal or antebrachiocarpal luxations, with limited description of the structures involved. This lack of detailed characterization underscores the relevance of the present findings, which provide experimental evidence for the differential stabilizing roles of individual carpal ligaments.

In the present study, in intact joints, extension, varus, and valgus ranges of motion showed no breed‐related differences. European shorthair cats had a mean varus–valgus range of 33.97°, similar to other breeds (34.34°). Extension averaged 26.47°, consistent with reports by Roos^1^ and Gonyea.^19^ Haut et al.^20^ reported age‐related variation in younger animals, but the present data suggest that carpal motion remains relatively stable once skeletal maturity is achieved.

Assessing carpal stability under controlled biomechanical conditions is technically challenging, particularly in small joints such as the feline carpus. Manual fixation during imaging allows rapid manipulation but does not provide standardized force application or reproducible load distribution, limiting its value for biomechanical analysis.

Previous studies in human and veterinary medicine have addressed these limitations using mechanically assisted or computer‐based systems.^21^, ^22^ However, the approaches described by Milgram et al.^14^ and Bitton et al.^23^ are not readily applicable to the feline carpus. They are typically limited to single‐plane motion, often require removal of stabilizing soft tissues, and do not allow controlled multiplanar loading.^2^ Radiographic assessment in live cats is also affected by physiological forelimb rotation and shoulder mobility, which hinder consistent positioning and load application.^24^

The experimental setup used in the present study was designed to overcome these limitations by enabling standardized, reproducible multiplanar loading while preserving relevant stabilizing structures and maintaining radiographic visibility. This allowed differentiation between physiological motion and pathological instability following selective desmotomy, although some restriction of normal limb motion was unavoidable.

Measurement reliability was supported by low error and high intertester agreement, comparable with previous radiokinematic studies.^17^, ^25^

Within this controlled framework, substantial interindividual variability in carpal range of motion was observed, consistent with previous reports.^24^ In contrast, left–right differences were minimal, supporting the use of contralateral limbs as internal controls.

Reported passive motion ranges in cats vary widely, from approximately 18° (varus–valgus)^26^ to 50°.^1^ The values measured in the present study (14.7°–50.9°, mean 34.12°) mostly fall within this range. Extension values (16°–42°, mean 26.47°) were comparable to previous reports.^1^, ^19^ This variability may reflect differences in habitual loading, as joint mobility has been linked to activity level in other species,^27^, ^28^, ^29^ although such data were not available in the present study.

Isolated transection of the medial collateral ligament did not produce radiographically detectable instability, although mild valgus deviation was observed in one specimen. This finding is consistent with feline data from Voss et al.^5^ but contrasts with canine studies by Wernitz,^9^ in which medial collateral injury resulted in marked instability, suggesting species‐specific differences in carpal stabilization. In cats, the palmar radiocarpal ligament appears to function as an important secondary stabilizer, as luxation of the proximal carpal row occurred only after combined transection of the medial collateral and palmar radiocarpal ligaments.

The stabilizing role of palmar structures was further supported by the marked hyperextension and valgus angulation observed after transection of the flexor retinaculum, whereas isolated tenotomy of the common digital flexor muscle did not affect passive stability. When the medial collateral ligament and flexor retinaculum were transected while the palmar radiocarpal ligament remained intact, extension increased, consistent with findings by Milgram et al.^14^ These results indicate a combined stabilizing effect of the medial collateral and palmar radiocarpal ligaments.

Inconsistent instability following isolated medial collateral ligament transection likely reflects the absence of dynamic muscular support and compensation by intact capsuloligamentous structures. This supports the concept that feline carpal stability depends on an integrated ligamentous network rather than a single dominant structure, as previously suggested by Roos.^24^

Dorsal carpal instability is uncommon in dogs^5^, ^29^, ^30^ and extremely rare in cats, with only a few reported cases.^9^, ^30^, ^31^ In dogs, luxation of the intermedioradial carpal bone has been associated with rupture of the medial collateral ligament in combination with the dorsal radiocarpal, capsular, and palmar ligaments, and may involve 90° rotation of the bone.^30^ Palierne et al.^32^ demonstrated, in a canine cadaver model, that this luxation can occur even with an intact medial collateral ligament.

In the present study, isolated or combined desmotomy of the dorsal radiocarpal and medial collateral ligaments did not result in measurable instability, suggesting that dorsal luxation of the feline antebrachiocarpal joint is unlikely without substantial palmar and intercarpal ligament disruption. However, experimental loading may not have fully replicated in vivo traumatic forces. Clinical reports of dorsomedial antebrachiocarpal luxation in cats managed without arthrodesis suggest that clinical luxation likely results from complex, multidirectional trauma involving additional soft‐tissue structures.^33^

Disruption of the lateral collateral ligament consistently caused marked instability of the ulnar aspect of the carpus, indicating that it serves as the primary stabilizer of the lateral compartment of the proximal carpal joint. In contrast, the ulnopalmar and accessorioulnar ligaments appeared to provide secondary support, as their isolated transection did not result in gross instability.

Stability of the accessory carpal joint appears to depend on integrated palmar ligamentous complexes rather than individual ligaments. Dense fiber integration among palmar intercarpal and carpometacarpal structures suggests that these ligaments function as a cohesive unit, making isolated failure unlikely to cause instability. Accessory carpal joint instability occurred only when ligamentous structures at the middle carpal level were disrupted.

Previous work in dogs has suggested an association between rupture of the accessory carpal–ulnar ligament and subluxation of the accessory carpal bone;^4^ however, this structure could not be macroscopically identified as a distinct stabilizing element in the present study, likely due to dense integration with surrounding palmar ulnocarpal and intercarpal ligamentous tissues. This supports the concept that accessory carpal joint stability depends on the integrity of palmar ligamentous complexes rather than isolated structures.

The absence of detectable instability following isolated or combined myotomy of the flexor digitorum communis, flexor carpi ulnaris, and flexor carpi radialis suggests that these muscles contribute little to passive stabilization of the feline carpus under static loading conditions, emphasizing the dominant stabilizing role of the palmar retinaculum and associated ligamentous structures.

Clinically, disruption of palmar carpal stabilizing structures has been associated closely with carpal hyperextension and instability in cats. Simpson and Goldsmid^34^ reported that feline carpal hyperextension injuries frequently require pancarpal arthrodesis to restore limb function. More recent clinical guidance suggests a more selective approach, with Basa et al.^8^ proposing primary ligament repair combined with rigid stabilization and external support in selected cases, reserving pancarpal arthrodesis for persistent instability or lameness. Calvo et al.^35^ reported generally good limb function following pancarpal or partial carpal arthrodesis in cats, although reduced jumping and climbing ability were noted. The present biomechanical findings help integrate these clinical observations by identifying the stabilizing structures responsible for maintaining carpal integrity.

Several limitations must be acknowledged. The cadaveric design excluded active muscle tone and in vivo neuromuscular dynamics. Repeated freeze–thaw cycles may have affected tissue integrity. Functional isolation of specific ligaments was sometimes impossible. Although the experimental design involved sequential transection of individual ligaments to assess their biomechanical contribution, ligament injuries in clinical trauma rarely occur in isolation and are typically accompanied by disruption of surrounding fascia, capsule, and other soft tissues. The experimental model cannot therefore fully replicate the complexity of clinical injury patterns.

An additional limitation relates to stabilization of the metacarpal bones during testing. Fixation of the metacarpi precluded assessment of distal instability and prevented evaluation of digital posture or collapse of the digits, which may be clinically relevant in cats with generalized muscular or connective tissue laxity. It also likely restricted excursion of the digital flexor and extensor tendons, whose coordinated gliding contributes to claw protraction, retraction, and load distribution across the carpus, potentially altering biomechanics under the experimental conditions.

Elimination of physiological radioulnar rotation by the fixation device represents another limitation. Although such motion occurs during normal feline locomotion, it was restricted to ensure reproducible loading and isolate ligament‐specific effects. The loading protocol focused on controlled flexion–extension and varus–valgus stresses, whereas traumatic carpal injuries in vivo likely involve multidirectional and torsional forces not reproduced in this setup. The sample size was limited, and variability in breed, age, and body size may have influenced joint flexibility. Although loading and positioning were standardized, minor differences in cerclage placement or imaging may have affected the results. Finally, interpretation remains challenging because of the scarcity of feline‐specific biomechanical data and the reliance on canine and human studies.

### 4.1 Conclusions

This study provides the first systematic biomechanical evaluation of feline carpal ligaments under standardized loading. Selective transection and stability testing identified the relative contributions of key stabilizers, showing that carpal stability relies on coordinated ligamentous and fascial interactions rather than a single dominant structure. These findings form a biomechanical basis for improving the diagnosis and treatment of feline carpal injuries.

## FUNDING INFORMATION

The authors received no financial support for this study.

## CONFLICT OF INTEREST

The authors declare no conflict of interest related to this report.

## Supporting information


**Figure S1:** Different angles from which the carpal joint bones were radiographed.
**Figure S2.** Cat forearm fixed in double‐prism holder (Künne, 2001). Left, relaxed; R, Radius; right, wing screws tightened; U, Ulna.
**Table S1.** Mean angular changes after selective desmotomy of feline carpal stabilizing structures. Δ, difference; Tol, Tolerance.
**Table S2.** Mean angular changes after selective desmotomy of feline carpal stabilizing structures with radiographical findings. Δ, difference; Tol, Tolerance.

## Data Availability

The data that support the findings of this study are available from the corresponding author upon reasonable request.
