# Supplementary material for: Biomechanical evaluation of carpal joint ligaments in the cat: An experimental and radiographic cadaveric study
Source: Vet Surg. 2026 Apr 28;55(7):1275–85. doi: 10.1111/vsu.70114 (PMC13643615; doi:10.1111/vsu.70114)
Supplement: Supplementary file 1 [file VSU-55-1275-s001.docx]

| **Ligament / Procedure** | **n** | **Varus (Pre / Post / Δ / p / Tol.)** | **Valgus (Pre / Post / Δ / p / Tol.)** | **Extension (Pre / Post / Δ / p / Tol.)** |
| --- | --- | --- | --- | --- |
| **Medial collateral ligament desmotomy** | 12 | 16.30° (SD 4.70) / 16.26° (SD 5.39) / −0.04° / p=0.81 / 3.50° | 20.35° (SD 8.40) / 22.92° (SD 9.06) / +2.57° / p=0.04 / 6.40° | 31.60° (SD 7.10) / 32.00° (SD 5.57) / +0.40° / p=0.66 / 6.00° |
| **Lateral collateral ligament (LCL) desmotomy** | 6 | 16.25° (SD 2.36) / 33.00° (SD 7.11) / +16.75° / p=0.02 / 3.50° | 20.21° (SD 4.56) / 19.93° (SD 3.50) / −0.28° / p=0.91 / 6.40° | 31.50° (SD 6.40) / 31.00° (SD 6.21) / −0.50° / p=0.70 / 6.00° |
| **Palmar ulnocarpal + accessory ulnar ligaments desmotomy** | 7 | 15.00° (SD 2.69) / 17.55° (SD 2.73) / +2.55° / p=0.01 / 3.50° | 18.98° (SD 6.20) / 19.80° (SD 5.10) / +0.82° / p=0.49 / 6.40° | 23.71° (SD 1.38) / 22.85° (SD 1.95) / −0.86° / p=0.18 / 6.00° |
| **Palmar radiocarpal ligament desmotomy** | 8 | 15.97° (SD 2.59) / 16.02° (SD 6.90) / +0.05° / p=1.00 / 3.50° | 17.90° (SD 5.22) / 25.07° (SD 4.85) / +7.17° / p=0.01 / 6.40° | 22.50° (SD 5.21) / 50.38° (SD 6.88) / +27.88° / p=0.01 / 6.00° |
| **Palmar intercarpal ligaments desmotomy** | 4 | 10.02° (SD 0.71) / 10.92° (SD 1.24) / +0.90° / p=0.06 / 3.50° | 14.60° (SD 2.28) / 15.97° (SD 6.15) / +1.37° / p=0.46 / 6.40° | 22.50° (SD 3.00) / 35.50° (SD 8.69) / +13.00° / p=0.06 / 6.00° |
| **Palmar carpometacarpal ligaments desmotomy (merged)** | 7 | 12.91° (SD 5.07) / 12.77° (SD 5.11) / −0.14° / p=0.49 / 3.50° | 21.28° (SD 2.88) / 23.65° (SD 2.71) / +2.37° / p=0.11 / 6.40° | 25.71° (SD 4.82) / 38.00° (SD 8.64) / +12.29° / p=0.01 / 6.00° |
| **Dorsal radiocarpal ligament desmotomy** | 4 | 13.60° (SD 6.64) / 14.45° (SD 8.71) / +0.85° / p=0.27 / 3.50° | 18.00° (SD 3.83) / 18.50° (SD 3.70) / +0.50° / p=0.85 / 6.40° | 24.50° (SD 4.43) / 25.50° (SD 5.26) / +1.00° / p=0.41 / 6.00° |
| **Medial collateral + dorsal radiocarpal ligaments (combined desmotomy)** | 4 | 13.60° (SD 6.64) / 14.32° (SD 6.87) / +0.72° / p=0.46 / 3.50° | 17.82° (SD 3.84) / 20.17° (SD 6.31) / +2.35° / p=0.14 / 6.40° | 24.50° (SD 4.43) / 25.00° (SD 4.76) / +0.50° / p=0.56 / 6.00° |
| **Accessoriometacarpal ligament desmotomy** | 2 | 10.55° / 13.20° / +2.65° / p=n/a / 3.50° | Mean Δ ≈ +1.60° / p=n/a / 6.40° | Mean Δ ≈ +5.00° / p=n/a / 6.00° |
| **Myotomy of flexor digitorum communis (FDC)** | 4 | 12.75° (SD 2.54) / 13.35° (SD 3.41) / +0.60° / p=0.58 / 3.50° | 15.87° (SD 0.88) / 16.20° (SD 3.37) / +0.33° / p=1.00 / 6.40° | 24.50° (SD 1.91) / 27.00° (SD 3.46) / +2.50° / p=0.10 / 6.00° |
| **Tenotomy of distal tendons of flexor carpi radialis + flexor carpi ulnaris** | 4 | 18.70° (SD 2.02) / 18.90° (SD 1.78) / +0.20° / p=1.00 / 3.50° | 15.45° (SD 2.74) / 15.22° (SD 3.97) / −0.23° / p=1.00 / 6.40° | 29.00° (SD 4.76) / 32.00° (SD 4.32) / +3.00° / p=0.08 / 6.00° |
| **FDC myotomy + tendon sheath + palmar retinaculum transection** | 6 | 12.75° / 13.35° / +0.60° / p=0.75 / 3.50° | 15.87° / 16.20° / +0.33° / p=0.29 / 6.40° | 22.66° (SD 3.72) / 41.66° (SD 6.74) / +19.00° / p=0.02 / 6.00° |
| **Medial collateral desmotomy + FDC myotomy + sheath + retinaculum transection** | 2 | — / — / — / p=n/a / 3.50° | 15.20° / 35.35° / +20.15° / p=n/a / 6.40° | 24.00° / 50.00° / +26.00° / p=n/a / 6.00° |

**Table 1:** Mean angular changes after selective desmotomy of feline carpal stabilizing structures Abbreviations: Δ: difference; Tol: Tolerance

| **Ligament/procedure** | **Specimens/Id** | **n** | **Varus (Pre / Post / Δ / p / Tol.)** | **Valgus (Pre / Post / Δ / p / Tol.)** | **Extension (Pre / Post / Δ / p / Tol.)** | **Radiographic Findings** | **Notes / Outliers** |
| --- | --- | --- | --- | --- | --- | --- | --- |
| **Medial collateral ligament desmotomy** | 3, 4, 5, 6, 17, 18, 23, 24, 39, 40, 55, 56 | 12 | 16.30° (SD 4.70) / 16.26° (SD 5.39) / −0.04° / p=0.81 / 3.50° | 20.35° (SD 8.40) / 22.92° (SD 9.06) / +2.57° / p=0.04 / 6.40° | 31.60° (SD 7.10) / 32.00° (SD 5.57) / +0.40° / p=0.66 / 6.00° | Neutral dorsopalmar views showed no misalignment, luxation, or subluxation in any specimen. | One outlier (ID 6) increased to 34.0° valgus, exceeding tolerance by 4.9°. |
| **Lateral collateral ligament (LCL) – desmotomy** | 1, 10, 29, 30, 31, 32 | 6 | 16.25° (SD 2.36) / 33.00° (SD 7.11) / +16.75° / p=0.02 / 3.50° | 20.21° (SD 4.56) / 19.93° (SD 3.50) / −0.28° / p=0.91 / 6.40° | 31.50° (SD 6.40) / 31.00° (SD 6.21) / −0.50° / p=0.70 / 6.00° | Subluxation of the ulnocarpal joint in varus in all specimens; in several, subluxation also in neutral position; in specimen 31, subluxation visible in extension as well. | Total DP range of motion increased from 34.12° (intact) to 52.83° post-desmotomy, exceeding the 9.9° measurement-error tolerance by 8.81°. |
| **Palmar ulnocarpal + accessory ulnar ligaments – desmotomy** | 9, 35, 36, 43, 44, 47, 48 | 7 | 15.00° (SD 2.69) / 17.55° (SD 2.73) / +2.55° / p=0.01 / 3.50° | 18.98° (SD 6.20) / 19.80° (SD 5.10) / +0.82° / p=0.49 / 6.40° | 23.71° (SD 1.38) / 22.85° (SD 1.95) / −0.86° / p=0.18 / 6.00° | Subluxation of the ulnocarpal joint observed in specimens 44, 47, and 48 (neutral and varus in 47–48; varus only in 44). | - |
| **Palmar radiocarpal ligament – desmotomy** | 25, 26, 33, 34, 37, 38, 59, 60 | 8 | 15.97° (SD 2.59) / 16.02° (SD 6.90) / +0.05° / p=1.00 / 3.50° | 17.90° (SD 5.22) / 25.07° (SD 4.85) / +7.17° / p=0.01 / 6.40° | 22.50° (SD 5.21) / 50.38° (SD 6.88) / +27.88° / p=0.01 / 6.00° | Consistent instability of the radiocarpal joint in valgus and extension: all limbs showed radiocarpal subluxation in valgus; all exhibited hyperextension with concurrent radiocarpal subluxation; in several, antebrachiocarpal hyperextension was also present; some limbs (IDs 25, 26) displayed radiocarpal subluxation already in neutral and/or varus. | Profound proximal carpal destabilization after transection. |
| **Palmar intercarpal ligaments – desmotomy** | 11, 12, 41, 42 | 4 | 10.02° (SD 0.71) / 10.92° (SD 1.24) / +0.90° / p=0.06 / 3.50° | 14.60° (SD 2.28) / 15.97° (SD 6.15) / +1.37° / p=0.46 / 6.40° | 22.50° (SD 3.00) / 35.50° (SD 8.69) / +13.00° / p=0.06 / 6.00° | Accessory-ulnar joint luxation across varus, neutral, and valgus in all specimens; mediocarpal joint subluxation present in neutral and valgus in most specimens; no new discrete structural failure identified in extension, although the joint was functionally hyperextended in all specimens. | Visualization required release of palmar soft tissues (see combined releases below). |
| **Palmar carpometacarpal ligaments – desmotomy (merged)** | 19, 20, 21, 53, 54, 57, 58 | 7 | 12.91° (SD 5.07) / 12.77° (SD 5.11) / −0.14° / p=0.49 / 3.50° | 21.28° (SD 2.88) / 23.65° (SD 2.71) / +2.37° / p=0.11 / 6.40° | 25.71° (SD 4.82) / 38.00° (SD 8.64) / +12.29° / p=0.01 / 6.00° | Hyperextension with carpometacarpal joint subluxation in most specimens (e.g., 19, 20, 21, 57), and complete carpometacarpal luxation in specimen 21; mediocarpal joint subluxation was common. | Source note mentions “specimen 44” lateral instability; 44 is not part of this group and is excluded from n (likely referenced from another intervention). |
| **Dorsal radiocarpal ligament – desmotomy** | 15, 16, 49, 50 | 4 | 13.60° (SD 6.64) / 14.45° (SD 8.71) / +0.85° / p=0.27 / 3.50° | 18.00° (SD 3.83) / 18.50° (SD 3.70) / +0.50° / p=0.85 / 6.40° | 24.50° (SD 4.43) / 25.50° (SD 5.26) / +1.00° / p=0.41 / 6.00° | Neutral dorsopalmar and mediolateral projections showed no misalignment, subluxation, or structural change before or after desmotomy. | - |
| **Medial collateral + dorsal radiocarpal ligaments – combined desmotomy** | 15, 16, 49, 50 | 4 | 13.60° (SD 6.64) / 14.32° (SD 6.87) / +0.72° / p=0.46 / 3.50° | 17.82° (SD 3.84) / 20.17° (SD 6.31) / +2.35° / p=0.14 / 6.40° | 24.50° (SD 4.43) / 25.00° (SD 4.76) / +0.50° / p=0.56 / 6.00° | Neutral dorsopalmar projections showed no radiographic signs of instability, subluxation, or structural change before or after desmotomy. | - |
| **Accessoriometacarpal ligament – desmotomy** | 41, 42 | 2 | 10.55° / 13.20° / +2.65° / p=n/a / 3.50° | (Δ: +2.60° and +0.60° individually) → mean approx. +1.60° / p=n/a / 6.40° | (Δ: +6.00° and +4.00° individually) → mean approx. +5.00° / p=n/a / 6.00° | No radiographic signs of malalignment or instability in any tested position (varus, neutral, valgus, extension). | Means approximated from individual values; authors state “no measurable instability.” |
| **Myotomy of flexor digitorum communis (FDC)** | 13, 14, 41, 42 | 4 | 12.75° (SD 2.54) / 13.35° (SD 3.41) / +0.60° / p=0.58 / 3.50° | 15.87° (SD 0.88) / 16.20° (SD 3.37) / +0.33° / p=1.00 / 6.40° | 24.50° (SD 1.91) / 27.00° (SD 3.46) / +2.50° / p=0.10 / 6.00° | No radiographic instability in neutral, varus, valgus, or extension. | - |
| **Tenotomy of distal tendons of flexor carpi radialis and flexor carpi ulnaris** | 27, 28, 45, 46 | 4 | 18.70° (SD 2.02) / 18.90° (SD 1.78) / +0.20° / p=1.00 / 3.50° | 15.45° (SD 2.74) / 15.22° (SD 3.97) / −0.23° / p=1.00 / 6.40° | 29.00° (SD 4.76) / 32.00° (SD 4.32) / +3.00° / p=0.08 / 6.00° | No signs of malalignment or instability in any projection. | - |
| **FDC myotomy + transection of its tendon sheath + palmar retinaculum** | 13, 14, 25, 26, 41, 42 | 6 | 12.75° / 13.35° / +0.60° / p=0.75 / 3.50° | 15.87° / 16.20° / +0.33° / p=0.29 / 6.40° | 22.66° (SD 3.72) / 41.66° (SD 6.74) / +19.00° / p=0.02 / 6.00° | Radiographs showed radiocarpal joint subluxation in specimens 13, 14, 25, and 26, with hyperextension involving the antebrachiocarpal joint; specimen 14 also displayed antebrachiocarpal subluxation in neutral; specimen 41 showed mediocarpal subluxation; specimen 42 showed generalized carpal hyperextension without a single focal subluxation. | Substantial loss of sagittal stability despite minimal coronal changes. |
| **Medial collateral desmotomy + FDC myotomy + sheath and retinaculum transection** | 13.14 | 2 | — / — / — / p=n/a / 3.50° | 15.20° / 35.35° / +20.15° / p=n/a / 6.40° | 24.00° / 50.00° / +26.00° / p=n/a / 6.00° | Radiocarpal joint subluxation in all positions with antebrachiocarpal hyperextension; specimen 14 additionally showed antebrachiocarpal subluxation in neutral. | Varus reported as slight decreases within tolerance; exact angles not specified in source text. |

Abbreviations: SD: Standard deviation; FDC: Flexor digitorum communis.Blue higlighting- significant

**Table 2**: Mean angular changes after selective desmotomy of feline carpal stabilizing structures with radiographical findings. Abbreviations: Δ: difference; Tol: Tolerance

**Figure 1**: Different angles from which the carpal joint bones were radiographed.

**Figure 2:** Cat forearm fixed in double-prism holder (Künne, 2001). U = Ulna, R = Radius; left = relaxed, right = wing screws tightened.
